# Influencing factors of information security behavior among college students based on protection motivation theory: evidence from China

**DOI:** 10.3389/fpubh.2025.1677024

**Published:** 2025-10-23

**Authors:** Meng Han, Haocun Zhao, Xiaohui Ma, Ruyi Shi

**Affiliations:** ^1^School of Economics and Management, China University of Mining and Technology, Xuzhou, China; ^2^Xuzhou Vocational Technology Academy of Finance & Economics, Jiangsu Union Technical Institute, Xuzhou, China; ^3^School of Public Policy & Management, China University of Mining and Technology, Xuzhou, China; ^4^BYD Auto Industry Company Limited, Shenzhen, China

**Keywords:** college student, protection motivation theory, information security behavior, threat appraisal, coping appraisal

## Abstract

**Introduction:**

With the advent of the information economy era, incidents of personal data breaches have occurred frequently, and the issue of personal information protection has become increasingly prominent. As primary users of Internet services, college students have seen their information security behavior emerge as a focal point of both academic inquiry and public concern. Investigating the factors influencing these behaviors holds substantial significance for enhancing the quality of university-based information security education and advancing the development of safe campus ecosystems.

**Methods:**

Based on the Protection Motivation Theory (PMT), this study constructed hypotheses about influencing factors of information security behavior among college students. Drawing on urban distribution across China, 23 cities were selected for data collection, with college students as the target population. A total of 3,030 valid questionnaires were ultimately retained. Data analysis was conducted by SPSS 20.0, including reliability tests, validity tests and regression analysis, to systematically explore the relationship between information security behavior and threat appraisal (perceived threat) as well as coping appraisal (self-efficacy, response efficacy and response cost).

**Results:**

Empirical analysis indicates that perceived threat, self-efficacy, and response efficacy exert a significant positive effect on college students’ information security behavior, among which response efficacy demonstrates the strongest positive impact. Conversely, response cost shows a significant negative impact on college students’ information security behavior.

**Discussion:**

These findings not only help enrich the knowledge system in the field of information security, but also provides practical insights for strengthening the campus information security environments. Furthermore, they provide actionable insights for policymakers tasked with addressing issues in information security behavior.

## 1 Introduction

AI applications (e.g., ChatGPT, Sora, DeepSeek) and privacy computing technologies have exponentially increased the exposure and potential impact of information threats, elevating the importance of research on information security behavior to an unprecedented level (1). In recent years, college students worldwide have faced growing risks to personal privacy and security. Once their personal information is leaked, it will be exploited by lawbreakers. A survey of over 60,000 Chinese college students found 44% reported sharing real personal information online, while 26% displayed excessive trust in the authenticity of online content (2). This issue is not unique to China, the official 2023 cyber security breaches survey of the UK shown that 61% of higher education institutions had suffered negative impacts (e.g., financial losses, data leakage) from security breaches or attacks, with this proportion standing at 36% for colleges (3). Driven by internet technologies, college students’ personal information can be leaked through multiple channels. Information asymmetry objectively weakens their ability to detect information security risks, leading them to lower vigilance. They may give up security protections or even participate in illegal information transactions, because it is difficult for them to recognize the hidden costs behind benefits. The 2023 Data Breach Investigations Report by Verizon posited that 74% of data breaches involve human factors (4). With the rise of digital technology, utilitarian nature of capitalism drives capital to transform personal information into a commodity. As a prime target, college students’ information and data are vulnerable to leakage and excessive collection. They often lack the ability to independently address information security incidents when they occur. Moreover, their high acceptance of new technologies leads to extensive engagement with cyberspace, making them a key group for understanding current and future trends in information security behavior. Safeguarding students’ information security and constructing an information security framework have become urgent and imperative tasks. This is not only a critical measure to prevent potential risks but also a vital foundation for keeping campuses safe and stable (5). Therefore, exploring the formation logic and influencing factors of college students’ information security behavior is essential to advancing practical campus-wide information security awareness education.

Driving factors of information security behavior exhibit multi-layered and multi-dimensional characteristics (6). Existing research primarily focuses on individual driven and environmental driven factors. Firstly, at the individual level, complexity spans dimensions of awareness, emotional responses, and motivation. Regarding awareness, studies indicate that entrepreneurs with stronger threat awareness usually have a more comprehensive understanding of cyber threats. But this may make them underestimate potential risks, consequently taking fewer protective measures (7). For emotional responses, this dimension includes a variety of psychological processes, including guilt, fear, and anger control mechanisms, all of which can influence individual information security behavior (8, 9). Positive emotional regulation enhances the stability and rationality of threat and coping appraisals, whereas negative emotions often prompt more conservative protective behavior choices (10). In terms of motivation, coping appraisal (e.g., self-efficacy, response efficacy) and threat appraisal are identified as key drivers of individuals’ intention to comply with cybersecurity norms (11). These two appraisals determine the intensity of individuals’ willingness to take protective behaviors (12). Secondly, at the environmental level, external contextual variables (e.g., informational environments, social norms, organizational systems) exert indirect effects on individuals. For the informational environment, cognitive resources are essential for triggering information security behavior. An individual may develop specific behaviors by selectively processing and recognizing the external environment and adjacent nodes (13). However, irrelevant or excessive information may distort threat appraisal and biases in coping appraisal, thereby undermining decision-making (14, 15). In the context of social norms, according to the Theory of Planned Behavior (TPB), an individual’s perceived social encouragement or pressure affects their compliance with or deviation from certain behaviors (16). Perceived social norms regarding compliance with Information Security Policy (ISP) have been found to significantly influence cybersecurity intentions (17). Additionally, punitive measures are typically used to strengthen information security behavior by enforcing and enhancing the cost of non-compliance and the cost of normative pressure (18). From the above literature, an analytical framework driven by individual and environmental factors has been established for the influencing factors of information security behavior. Compared with existing ISP compliance studies which mainly focus on environmental driven factors, this research limits its focus to the college student group, and conducts an in-depth study on college students’ driven factors of information security behavior from the perspective of individual internal attributes.

Scholars have widely applied the Protection Motivation Theory (PMT) to research on information security behavior-related issues. The PMT has been deployed in the study of information security behavior to understand how the perception of threat severity and vulnerability contribute to the motivational impetus to adopt protective behaviors (19). A survey conducted at Western Michigan University applied PMT to explore privacy protection behaviors among social networking site users (20). Similarly, another study extended the PMT model to address social networking site users’ privacy and security issues, revealing that response efficacy and individual responsibility were the most critical predictors of online safety intentions (21). Alrawhani et al. (11) found that self-efficacy, response efficacy, and perceived severity significantly influenced employees’ intention to comply with information security policies by using the protection motivation theory (13). In the context of emerging technology applications, Zhang (22) incorporated privacy concerns as a mediator into the PMT model, showing that perceived severity and response efficacy positively impacted privacy concerns, which in turn contributed to resistance against. A recent study examining entrepreneurs’ security behavior against ransomware employed an extended PMT model and extended it with subjective norms, threat awareness and affective response (9). It is found that existing studies on information security behavior on the basis of the PMT have mostly focused on social networking site, corporate employees and specific professional groups. However, college students differ fundamentally from entrepreneurs (with mature risk assessment capabilities) and corporate employees (subject to information security policy constraints) examined in existing studies. Within the PMT framework, the internal mechanisms influencing college students’ information security behavior remain underexplored (23). Furthermore, while the role of response efficacy in shaping behavioral intentions has been validated across multiple fields, it presents an opportunity for deeper investigation within the realm of college students’ information security (24). Similarly, although the impact of response cost on behavioral intentions has attracted academic attention (25), existing studies that integrate college students’ group-specific traits remain insufficient. These research gaps are more obvious when considering college students’ individual characteristics and unique information security behavioral patterns in the digital era.

Against this backdrop, this study intends to systematically investigate the factors influencing college students’ information security behavior based on the PMT model, thereby helping address the aforementioned research gaps. The study attempts to answer the following questions: What factors influence college students’ personal information security behavior in the digital age? What measures can effectively safeguard college students’ personal information security? To address these questions, this study formulated a PMT framework and built a testing model to explore the specific impacts of the four dimensions of protection motivation (perceived threat, self-efficacy, response efficacy, and response cost) on the college students’ information security behavior. On this basis, the study proposes countermeasures to mitigate potential risks to college students’ personal information security. In practice, this work offers evidence-based guidance for colleges and policymakers to develop targeted educational interventions and preventive strategies, ultimately reducing threats to students’ personal information security.

The structure of this paper is as follows: Section 2 elaborates on the PMT model as the theoretical foundation and puts forward research hypotheses; Section 3 describes the research methodology, including sample selection and measurement tools; Section 4 presents the results of reliability and validity tests for questionnaire items as well as the outcomes of hypothesis testing; Section 5 discusses the research findings in depth and puts forward corresponding countermeasures and suggestions; Section 6 summarizes the conclusions and implications of the study; Section 7 presents the limitations of this study and suggestions for further research.

## 2 Theoretical basis and research hypotheses

### 2.1 Theoretical basis

Protection Motivation Theory (PMT), developed by Rogers (26) in 1975, focuses on fear appeals to explain individuals’ engagement in protective behaviors. The theory has garnered broad recognition for its practical utility in the field of information security and is widely regarded as a robust framework for interpreting users’ information security-related behaviors (27). Its components align closely with the conceptual scope of information security, providing a rational theoretical foundation for research and yielding substantive findings (14).

**Figure 1:**
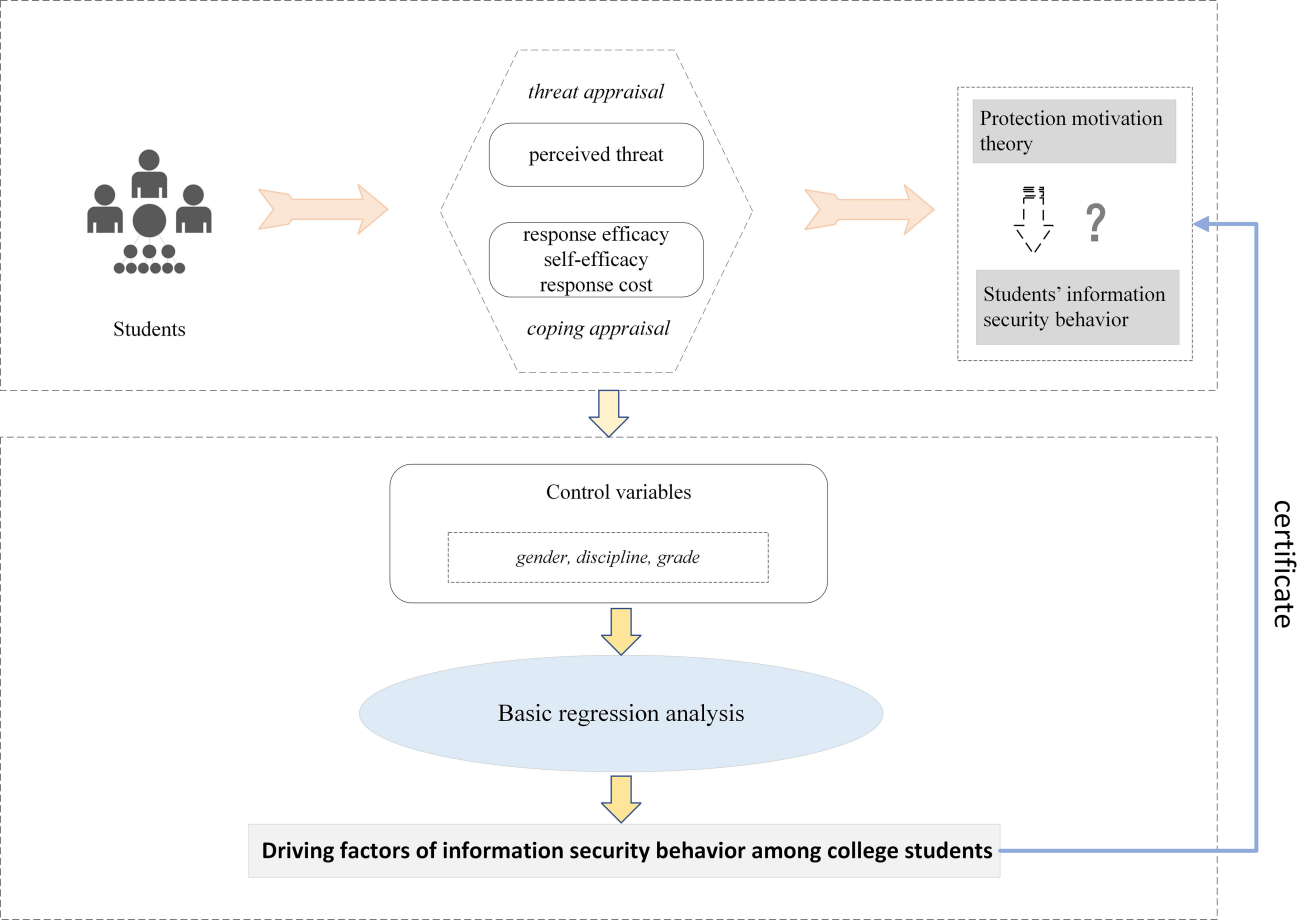
Research framework for the driving factors of college students’ information security behavior from the perspective of PMT.

PMT integrates threat appraisal and coping appraisal to explain the process of behavioral change. Threat appraisal reflects individuals’ perceptions of the likelihood of harmful outcomes arising from hazardous factors, while coping appraisal captures the balance between taking protective actions to avoid harm and alternative strategies (14). In practical terms, it means individuals should recognize both the likelihood of encountering a threat and the potential severity of its consequences. Concurrently, individuals need to acknowledge their capacity to implement protective behaviors to mitigate threats and judge whether the benefits of these actions outweigh their associated costs. Both threat appraisal and coping appraisal create intrinsic motivation, which in turn drives the adoption of protective behaviors. Threat appraisal includes perceived severity and perceived susceptibility (28). Perceived severity refers to the anticipated degree of harm when a threat materializes, whereas perceived susceptibility is the perceived probability of experiencing harmful events. Higher levels of perceived threat severity and susceptibility generate stronger protective motives, thereby facilitating protective behaviors. Coping appraisal includes response efficacy, self-efficacy, and response cost. Response efficacy shows how an individual evaluates the effectiveness of recommended protective measures. Self-efficacy denotes individuals’ confidence in their capacity to execute risk-prevention actions. Response cost refers to the psychological or physical burdens that come with executing control measures (29). Based on the above analysis, the research framework depicted in Figure 1 was constructed.

### 2.2 Research hypotheses

In the field of information security, information leakage and misuse are widely seen as direct threats to the personal property security of information subjects. In this situation, vulnerability refers to the perceived likelihood of a threat occurring or an individual encountering it. Users’ actions to protect their personal information depend on both information risk assessment and information response assessment (29). The former involves users’ judgments of the perceived intensity of information security threats, including the severity of potential harm and the likelihood of occurrence (30); the latter covers users’ evaluations of their ability to prevent risks. Self-efficacy denotes individuals’ capacity to implement personal information protection measures. Response efficacy shows the confidence that security protection actions can effectively get rid of information threats (31). Response cost is any expense related to carrying out information security behavior (32). It might appear as financial costs, time spent, or inconvenience caused when dealing with information security incidents. If these costs outweigh the perceived severity of the threat to individuals, they may choose not to address the threat after conducting a cost–benefit assessment.

Perceived threat is defined as the degree to which individuals perceive information leakage as dangerous or harmful (33). When individuals perceive these risks as insufficiently severe, they tend to reject risk protection strategies. This makes them more likely to be affected by risky behaviors. Perceived threat consists of threat severity and threat vulnerability. In the context of information risks, threat vulnerability means a person’s subjective assessment of the probability of suffering negative impacts from information leakage. Threat severity refers to their perceived seriousness of the negative consequences caused by information leakage (34). Internet usage capabilities are closely linked to risk awareness. Having a clear understanding of vulnerability and severity is very important. Previous studies on computer security have found that there is a strong positive connection between threat vulnerability and the intention to carry out information security behavior (31). Similarly, threat severity has been found to be significant in influencing the intention to use smart home devices (35). Therefore, there is a close association between college students’ perceived threat and their decision to use information security behavior (34). Specifically, the more serious college students think the risks to their personal information security are, the stronger their willingness is to use information security behavior to avoid those risks. Consequently, this study puts forward the following hypothesis:

*H1:* Perceived threat will positively influence students’ information security behavior.

Self-efficacy is defined as students’ confidence in their ability to implement safeguarding measures and it is a key determinant of protection motivation. In the context of information security, such safeguarding measures often include protection behaviors such as installing antivirus software, avoiding visits to illegal websites, and setting strong, unique passwords. In practice, individuals usually assess their own ability to support a specific action before deciding to take that action. Prior research has shown that as students’ self-efficacy increases, their motivation to engage in information security behavior also increases (36). Therefore, the higher students’ self-efficacy is in implementing safeguarding measures, the stronger their motivation will be to avoid information threats through those measures. Consequently, this study hypothesizes that:

*H2*: Self-efficacy will positively influence students’ information security behavior.

Response efficacy refers to an individual’s subjective assessment of the effectiveness of protective measures in preventing information threats. It reflects students’ perceptions of the objective outcomes of using such measures and aligns with the concept of outcome expectancy (37). If users believe that protective measures they adopt are effective and capable of yielding positive impacts, they are more inclined to take such actions proactively. For instance, when using online services, many users either refrain from using antivirus software or only occasionally scan their devices with pirated versions. It leaves their personal information vulnerable to potential threats. Research has indicated that using effective technical tools and implementing necessary safety measures significantly reduces the risk of information security breaches (38). Users who fully acknowledge the benefits of these tools and measures are more likely to proactively adopt positive protective behaviors (39). Prior information security studies have consistently suggested that response efficacy motivates students to engage in security behavior (40). If college students believe their proactive actions can significantly reduce the risk of information leakage, they will gradually increase their initiative to adopt information security protection measures. Therefore, this study proposes the following hypothesis:

*H3*: Response efficacy will positively influence students’ information security behavior.

In the context of information security, response cost is defined as all burdens associated with engaging in protective behaviors. This includes time investment, financial costs, mental effort, inconvenience, or even negative experiences. These costs act as barriers to action and diminish incentives for engaging in such behaviors, as individuals typically conduct a cost–benefit assessment prior to acting. When individuals perceive that the response costs for information security protection outweigh the expected benefits, they are less likely to implement specific protective behaviors. This perspective is supported by research in the field of personal computer usage, where higher perceived response costs were found to negatively predict willingness to engage in protective behaviors (41). Similarly, in the field of mobile wallet and banking usage, higher perceived response costs also deter individuals from adopting information security behavior (42). This study suggests that college students may need to spend certain amounts of time, energy or money when they engage in information security behavior. These expenses make up their response costs and affect how likely they are to take part in such behaviors. As such, this study hypothesizes that:

*H4*: Response cost will negatively influence students’ information security behavior.

**Figure 2:**
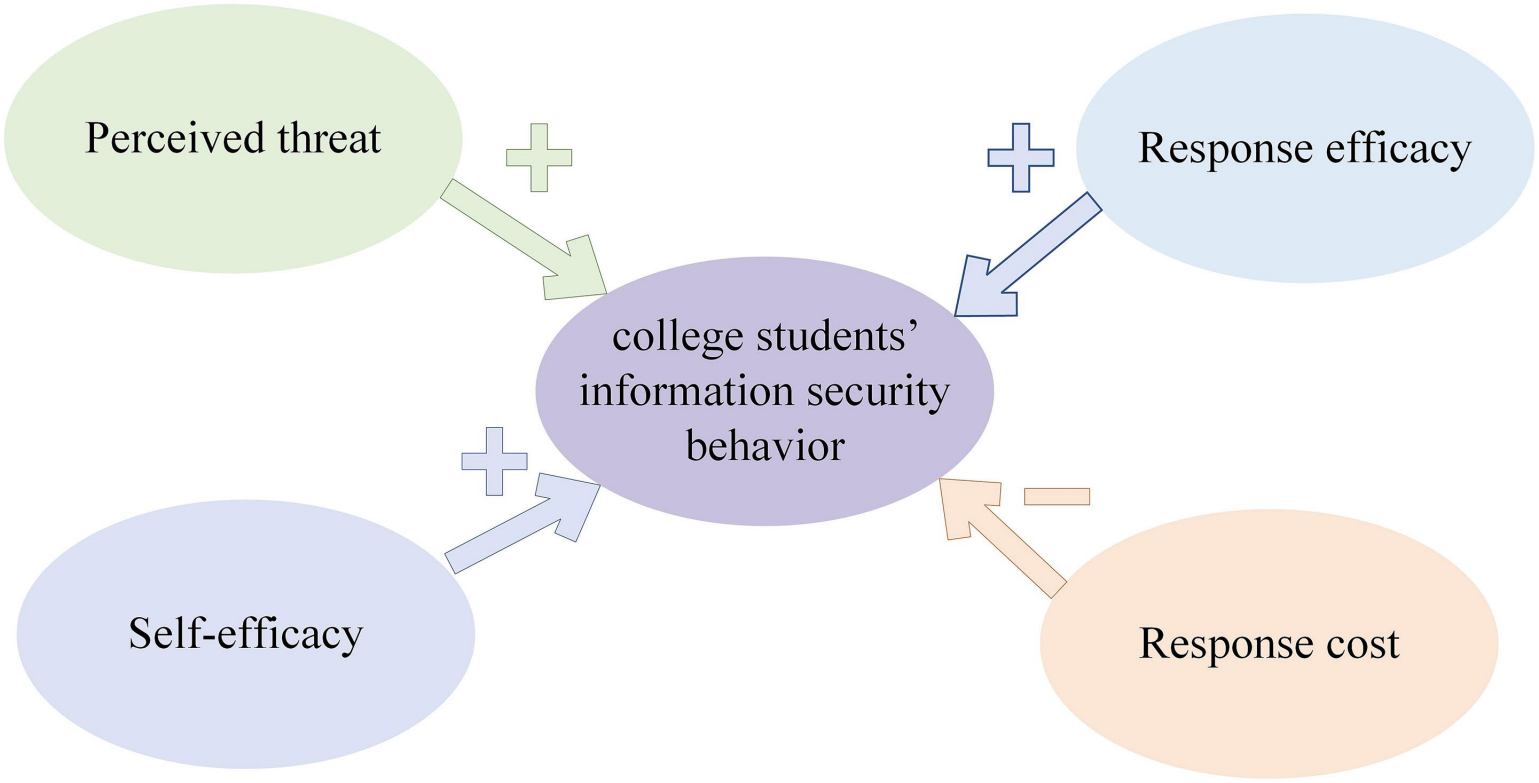
Research model of college students’ information security behavior.

Based on the above research hypotheses H1 to H4, the research model constructed in this study is presented in Figure 2.

## 3 Research methodology

### 3.1 Measurements

This study first conducts a comprehensive review of relevant literature to synthesize existing research on information security behavior. Building on PMT, we compiled related research variables and measurement items, integrated the hypotheses proposed in this study, and designed a questionnaire to investigate factors influencing college students’ information security behavior. The questionnaire includes five variables: perceived threat (PT) (41), self-efficacy (SE) (43), response efficacy (RE) (44), response cost (RC) (45), and information security behavior (ISB) (46), each measured by five items. It also contained four items capturing respondents’ demographic information. To further explore the barriers to college students’ information security behavior, an open-ended question was included, asking students to identify reasons they refrain from adopting information protection measures, such as setting strong passwords, installing antivirus software, and handling information carefully in their daily Internet use. All measurement items are derived from existing domestic and international literature, and the scale was adjusted according to the characteristics of college students’ information security behavior, resulting in the final research questionnaire. The questionnaire adopts a 5-point Likert scale for measuring variables, facilitating both data collection and subsequent analysis. Response options ranged from “1″ for “strongly disagree” and “5″ for “strongly agree.” Respondents answer the questionnaire based on their actual situations and with reference to their daily experiences. A preliminary study involving 230 participants from the target college student population was conducted to refine the questionnaire and ensure its clarity and relevance. For preliminary scale validation, the recommended sample size is three to five times the maximum number of items in any subscale. A larger sample size typically improves scale testing outcomes. While a minimum sample size of 15 is sufficient for preliminary assessments, the sample size for this study exceeded this threshold, meeting scientific research standards (47). Specific details regarding measurement indicators, their source literature, and item descriptions are presented in Table 1 below.

**Table 1:** Specific details of measurement indicators for college students in information security behavior research.

Variable	Items
Perceived threat (41)	There is a risk of my information security being leaked.
	The risk of my information security being leaked is relatively high.
	The leakage of information security will cause me certain losses.
	The leakage of information security will have a serious impact on my life.
	If information security causes serious impacts on my life, it will make me inclined to take information security measures.
Self-efficacy (43)	I know how to protect the security of my personal information.
	It is easy to take information security preventive measures.
	I can take measures to prevent others from infringing upon my personal information.
	I can help others protect their personal information.
	If I have sufficient knowledge and ability, I am willing to carry out information security behavior.
Response efficacy (44)	Taking preventive measures can effectively protect the security of personal information.
	Protecting the security of personal information through related technology is very effective.
	The efforts I can make to protect information security are effective.
	The success rate of taking actions to protect information security is extremely high.
	If carrying out information security behavior is beneficial, I will carry out it.
Response cost (45)	It is a bit troublesome to use information security measures.
	Carrying out information security behavior consumes my time and energy.
	I think that carrying out information security behavior will affect the convenience of my life.
	Since implementing information security measures incurs costs, it will have a serious impact on me.
	If a large amount of cost is incurred due to carrying out information security behavior, I will no longer be willing to carry out it.
College students’ information security behavior (46)	I am very willing to take some actions to protect the security of personal information.
	I will actively use information security technology in my daily life.
	I will actively learn information security knowledge in my daily life.
	I will not install unreliable application software on my electronic devices.
	I will not casually disclose my personal information in my daily life.

### 3.2 Data collection

This study collected data through a questionnaire survey, and the target group for questionnaire distribution was online college students. Participants in the survey had diverse backgrounds in terms of age, gender, and educational attainment. According to the urban distribution in various regions of China, 23 cities were selected, with 150 questionnaires distributed in each city. The sampling cities were as follows: two cities in northeastern China (Harbin and Dalian), six cities in eastern China (Shanghai, Nanjing, Hangzhou, Hefei, Xuzhou and Fuzhou), four cities in northern China (Beijing, Tianjin, Shijiazhuang and Taiyuan), three cities in central China (Wuhan, Zhengzhou, and Changsha), three cities in southern China (Guangzhou, Shenzhen, and Nanning), three cities in southwestern China (Chengdu, Kunming, and Chongqing), and two cities in northwestern China (Xi’ an and Lanzhou). Participants had been provided with clear and detailed information about the purpose of our study, the type of the questions, and how their responses would be used. Each participant had to read the informed consent file and agree to participate in the study before they could start the survey. It is important to note that, due to privacy concerns, the study did not collect data related to individuals’ mental health or other substance use. All participants’ responses had been treated as confidential and would not be disclosed to third parties.

The questionnaire survey was conducted online through three channels: personal networks, social media platforms such as WeChat, and a professional questionnaire website called Sojump. Sojump, the largest professional questionnaire platform in Chinese mainland in terms of user scale, serves as the core hub platform. It comes with built-in functions including logical jump, time limit for completion and IP address deduplication. These functions can block repeated submissions from the same IP address and invalid questionnaires that take an excessively short time to complete. We have stratified by administrative regions, with samples covering 23 cities across China; however, cross-city field surveys were difficult due to dual constraints of budget and team manpower. By contrast, the sample referral mechanism of snowball sampling can quickly reach target groups in various regions based on initial samples, effectively resolving the conflict between geographical dispersion and limited resources. It’s a feasible solution determined through repeated trade-offs between the research objective of ensuring sample diversity and the objective reality of limited human and financial resources. In practical operation, we have strictly controlled the sample size of snowball sampling. At the same time, to effectively reduce the sample bias that may be caused by non-probability sampling, we further designed and implemented several bias control strategies. Firstly, in terms of geographical and disciplinary coverage, representative cities were selected based on China’s seven major geographical divisions, covering different types of universities. This ensures that the disciplinary distribution is basically consistent with the disciplinary structure of Chinese universities and avoids geographical or disciplinary concentration of samples, thus ensure the sample diversity of our research (48). Secondly, in terms of process control for snowball sampling, the initial respondents were strictly selected to cover different grades and disciplines. These respondents were required to recommend peers who met the research subject criteria and were not in the same class or dormitory. Additionally, when the sample proportion of a certain geographical division or discipline reaches the preset target, the recommendation for that group is stopped to prevent excessive sample closure. Finally, we examined the representativeness of the sample relative to the target population. Specifically, non-graduating students accounted for a high proportion (93.27%) of the sample in this study, and this result is consistent with the research data reported by Zhan et al. (49). They conducted a survey on 1,586 students in China, which found that 96.87% of the respondents were non-graduating students. Moreover, freshmen and sophomores accounted for 44.82% of the sample in this study, and this proportion is also consistent with the findings of Wu et al. (50). They carried out a nationwide survey involving 11,954 college students in China, whose data showed that freshmen and sophomores accounted for 41.4% of the respondents. There was no significant difference between the sample structure of this study and the overall disciplinary structure and grade distribution of Chinese colleges and universities. This indicates that the sampling framework adopted in this study effectively reduced selection bias and ensured the representativeness of the sample (51).

The data collection phase began in June, and questionnaire data filled out by college students was collected by August 31. Taking advantage of college students’ sufficient time and high willingness to participate during the summer vacation, this study conducted questionnaire data collection among Chinese college students through multiple channels. To enhance their enthusiasm for participation, the structure of questionnaire was designed to be concise and easy to understand, with simple operational steps required for completion. Additionally, cash incentives were provided to respondents who completed valid questionnaires, and all target participants included in the study finished the questionnaire. A total of 3,651 questionnaires were collected; however, those with a response time of less than 50 s and those with obviously inconsistent responses were excluded, with 3,030 valid questionnaires finally retained. After verification, the demographic characteristics of the sample in this study showed good consistency with those of the overall population of college students nationwide. According to the relevant formula, with a common 5% sampling error and a 95% confidence level, the minimum required sample size is 385 valid questionnaires (52), and the sample size of this study meets the standard. As shown in Table 2, among the valid samples, 55.81% were female. From a grade distribution perspective, freshmen accounted for 18.12%, sophomores for 26.7%, juniors for 18.12%, and seniors for 30.33%. The proportion of undergraduate students across all grades was relatively balanced, while that of graduate students was lower. However, overall, the sample data spanned a wide range of academic disciplines, providing some representativeness in subsequent analyses. In terms of discipline categories, engineering and technical fields made up 59.14%. This was followed by humanities, social sciences and management, which accounted for 29.7%. Art and physical education made up a small proportion at only 2.94%. The study’s sample covered all academic disciplines and was broad, this diversity makes subsequent analytical studies both valuable and meaningful.

**Table 2:** Description of the distribution of sample characteristics.

Items	Frequency	Percentage	Items	Frequency	Percentage
Grade			Discipline		
Freshmen	549	18.12%	Humanities, social sciences, and management	900	29.7%
Sophomore	809	26.7%	Science and engineering	1792	59.14%
Junior	549	18.12%	Art and physical education	89	2.94%
Senior	919	30.33%	Others	249	8.22%
Graduate students	204	6.73%	Gender		
			Male	1,339	44.19%
			Female	1,691	55.81%

After organizing and cleaning the valid sample data, descriptive statistical analysis was performed to examine respondents’ attitudes and perceptions, using SPSS 20 software. The results showed that the mean scores of the four factors influencing college students’ information security behavior followed this order perceived threat at 3.98, response efficacy at 3.81, self-efficacy at 3.19 and response cost at 3.07. A higher mean score indicates a stronger recognition of these factors among college students. The average score of each of the four factors is greater than 3, which indicates that the sample respondents generally recognize the importance of these factors. The standard deviation results indicated relatively consistent opinions among respondents, with no significant disparities.

## 4 Results

### 4.1 Reliability and validity

To conduct factor analysis, this study first carried out the KMO test and Bartlett’s test of sphericity on the sample data. The KMO index was 0.891, which shows the data were suitable for factor analysis. Bartlett’s test of sphericity produced a significant result, confirming that there was a strong correlation among the variables, so factor analysis was also suitable to be conducted. Subsequently, the study tested the reliability and validity of the questionnaire. Factor loadings which are shown in Table 3 measure the correlation between observed variables that define the same latent variable, serving as an indicator of the measurement model’s convergent validity. Generally, factor loadings of at least 0.3 are considered acceptable, values greater than 0.5 are satisfactory, and those exceeding 0.7 are deemed excellent (53). Reliability and validity were evaluated using Cronbach’s alpha, average variance extracted (AVE), and composite reliability (CR). Criteria stipulate that Cronbach’s alpha or CR values should exceed 0.7, and AVE values should be greater than 0.5 (54). In this study, the Cronbach’s alpha coefficients of all variables were greater than 0.7, indicating that the variables had good internal consistency reliability. The standardized factor loadings of each item were greater than 0.5, the CR values of each construct were greater than 0.7, and the AVE values were greater than 0.5. All these met the criteria for convergent validity, and the model fit was also within the acceptable range. Therefore, all items were retained for subsequent analysis.

**Table 3:** Results of questionnaire credibility and reliability analyses.

Measurement variables	Item loadings	Mean	Cronbach’s α	AVE	CR
Perceived threat					
PT1	0.851	0.841	0.906	0.6943	0.9190
PT2	0.851	0.961			
PT3	0.857	0.876			
PT4	0.822	0.971			
PT5	0.783	0.847			
Self-efficacy					
SE1	0.711	0.973	0.893	0.6522	0.9019
SE2	0.890	0.936			
SE3	0.915	0.967			
SE4	0.859	1.016			
SE5	0.623	0.969			
Response efficacy					
RE1	0.831	0.821	0.911	0.5797	0.8719
RE2	0.836	0.771			
RE3	0.795	0.794			
RE4	0.605	0.883			
RE5	0.715	0.799			
Response cost					
RC1	0.831	0.978	0.916	0.7410	0.9346
RC2	0.881	1.028			
RC3	0.880	1.007			
RC4	0.897	1.015			
RC5	0.812	1.089			
Information security behavior					
ISB1	0.791	0.814	0.919	0.6496	0.9026
ISB2	0.813	0.845			
ISB3	0.825	0.801			
ISB4	0.784	0.876			
ISB5	0.816	0.810			

### 4.2 Explanatory analysis of information security behavior

Table 4 shows the Pearson correlation analysis between key variables and information security behavior. Perceived threat, self-efficacy, and response efficacy all had a significant positive correlation with the information security behavior index at the 1% significance level. This suggests that improvements in these variables may boost information security behavior. Among them, response efficacy exhibited the strongest correlation with information security behavior. It is indicated that information security behavior was positively correlated with perceived threat, self-efficacy, and response efficacy, while negatively correlated with response cost. The specific nature of these relationships requires further examination through multiple regression analysis to verify their statistical significance and theoretical implications.

**Table 4:** Pearson correlation analysis between major variables and information security behavior.

	Perceived threat	Self-efficacy	Response efficacy	Response cost
β	0.529*	0.430*	0.647*	−0.166*
P	0.000	0.000	0.000	0.004
N	3,030	3,030	3,030	3,030

*p < 0.001.

The hypotheses predicted that perceived threat (H1), self-efficacy (H2), response efficacy (H3), response cost (H4) would significantly predict security intentions. Regression analysis using SPSS 20 revealed the following significant effects on college students’ information security behavior (Table 5). Response efficacy had a significant positive impact (*β* = 0.470, *p*<0.001); perceived threat also exerted a significant positive effect (*β* = 0.289, *p*<0.001); response cost showed a significant negative influence (*β* = −0.156, *p*<0.001); and self-efficacy exhibited a marginally significant positive association (*β* = 0.092, *p*<0.05). The results show that perceived threat, response efficacy, and self-efficacy all exert a significant positive impact on college students’ information security behavior. This indicates that the more acute college students’ perception of information security risks is, the greater their trust in the effectiveness of information security measures, and the more confident they are in their own implementation capabilities, the more likely they are to adopt information security behavior.

**Table 5:** Results of regression analysis on college students’ information security behavior.

Hypotheses	Variable	Coefficients	Confirmed	R^2^	F(Sig)
H1: +	Perceived threat	0.289**	Yes	0.523	81.529 (0.000)
H2: +	Self-efficacy	0.092*	Yes		
H3: +	Response efficacy	0.470**	Yes		
H4: −	Response cost	−0.156**	Yes		

*p < 0.05, **p < 0.001.

Among these factors, response efficacy exerts a more prominent impact on college students’ information security behavior. The possible reason is that response efficacy plays a crucial role in the process of transforming perceived information threats into information security behavior (55). When college students fully recognize the actual effectiveness and potential benefits of such protective tools and measures, their tendency to adopt standardized information security behavior will be significantly enhanced (41). Additionally, the regression coefficient of response cost is significantly negative. This result reveals the restrictive effect of response cost on college students’ information security behavior. Specifically, the higher the cost, the more difficult it is to implement these behaviors. Hypotheses 1–4 are thus verified.

## 5 Discussion

**Figure 3:**
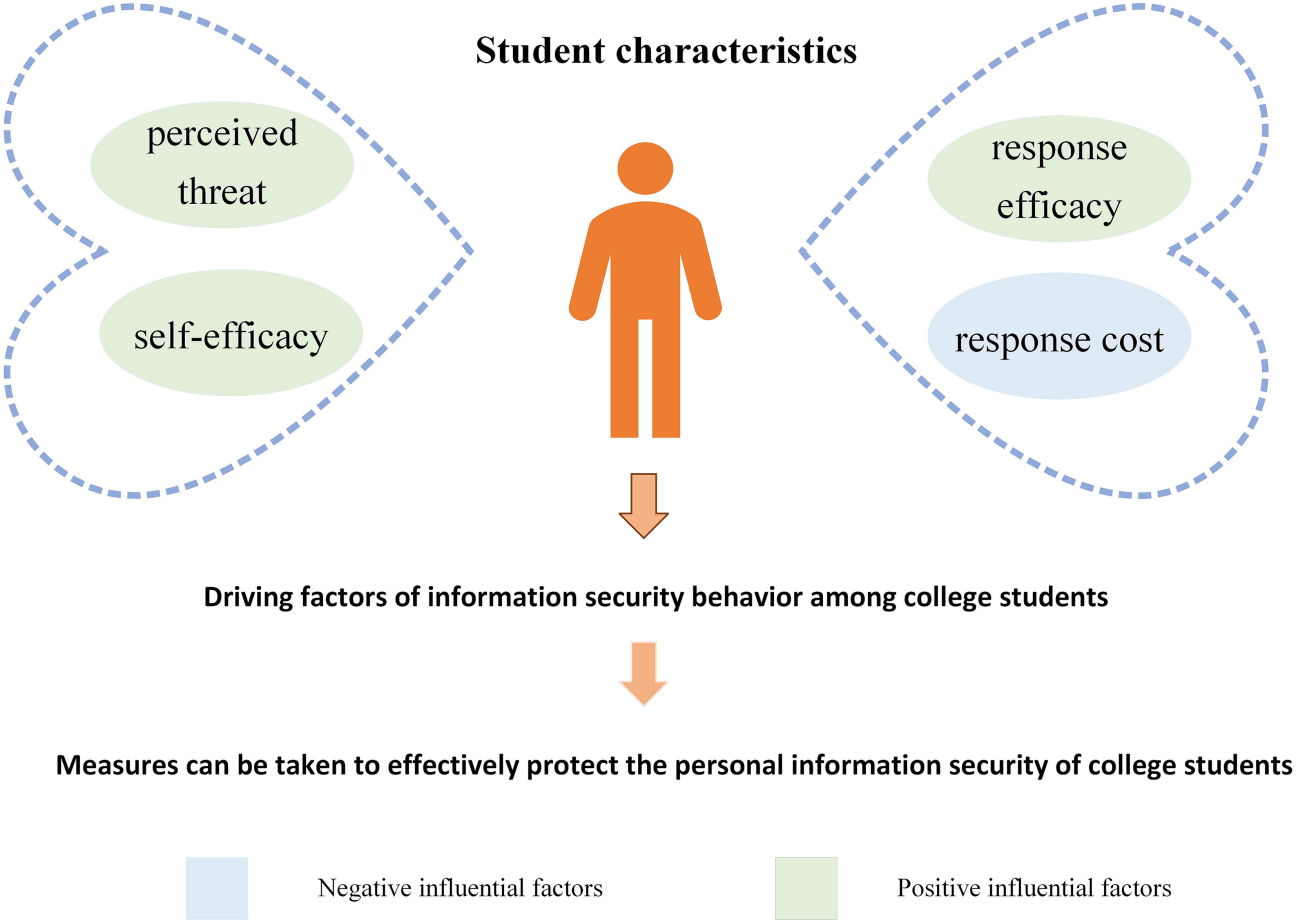
Driving factors of college students’ information security behavior.

This study explores how perceived threat, response efficacy, self-efficacy and response cost impact college students’ information security behavior. The findings align with existing studies in the academic community. As shown in Figure 3, students’ perceived threat has a significant influence on their information security behavior, indicating that students’ awareness of potential threats directly shapes their actions to safeguard personal information. This result matches numerous previous studies around the world. Öğütçü (56) found that higher perceived threat levels among users correlate with more proactive protective behaviors. Mousavi et al. (20) not only verified that perceived threat is positively correlated with protection motivation, but also pointed out that perceived threat can negatively affect coping appraisal via privacy concerns. The development of protection motivation will further encourage users to adopt protective measures such as customizing privacy settings and discourages risky behaviors like personal information disclosure (21). The global relevance of this finding is further supported by Chen et al. (57), who compared Chinese and American users’ responses to network security threats and found that threat severity exerts a far stronger influence on Chinese users than on American users. Students with a stronger awareness of such threats tend to take the initiative to prevent unauthorized access to or modification of their personal information. Specifically, higher perceived threat increases students’ concern for privacy and security, which in turn drives their adoption of corresponding protective measures. Given the increasing sophistication and prevalence of personal information theft methods, students face diverse potential risks. Additionally, internet has inherent traits like anonymity, easy access, and built-in risks. These not only expose students to privacy breaches but may even threaten their property or physical safety in extreme cases. Consequently, governments and organizations must strengthen information security and privacy training programs. Universities should organize lectures and workshops focusing on information security incident cases to guide students in identifying and addressing potential threats. Additionally, students themselves should actively engage in learning and applying relevant knowledge. They should develop crisis prevention awareness while taking appropriate self-protection measures.

Students’ self-efficacy also exerts a significant positive impact on their information security behavior. While the magnitude of this effect is smaller than that reported by Chang et al. (58), it still contributes to students’ intentions to protect information privacy. This difference may stem from differences in sample characteristics. Beyond Chinese-specific contexts, prior research has found that when facing threats, Chinese users are more inclined to seek help, and that their self-efficacy has a greater impact on protective behaviors (21). However, our findings reveal that self-efficacy has a relatively weak impact on Chinese college students’ adoption of information security behavior. This may be attributed to the fact that as digital natives, college students engage frequently with the Internet and smart devices during their growth, fostering proficiency in basic information security practices. For Chinese college students, this proficiency translates to uniformly high levels of self-efficacy, with minimal variability across individuals, which weakens its explanatory power for behaviors. Furthermore, the phenomenon of information asymmetry tends to make college students develop optimistic bias (59). Optimistic bias refers to college students’ tendency to believe that they will not experience information security threats and to estimate the consequences of such threats more optimistically than the actual situation (60). This bias hinders the transformation of self-efficacy into actual information security protection behavior. Specifically, optimistic bias creates a cognitive illusion that risks are irrelevant to themselves, depriving the ability confidence embodied in self-efficacy of the motivational premise required for transforming into behaviors. Students’ self-efficacy mainly appears in their confidence to master and use operational skills flexibly, understand Internet safety protocols, and accurately identify and respond to diverse security threats. In our survey, when asked about barriers to information protection, some college students noted: *“I did not set a strong password because I thought I might easily forget it, and the subsequent password recovery process is a little tedious.”* This indicates that when college students lack confidence in the supporting abilities required to implement information security behavior such as remembering passwords and handling the password recovery process, they may avoid taking such actions even if they are aware of their importance. Higher self-efficacy levels are linked to stronger motivation to protect information privacy. Therefore, universities should organize information security and privacy education initiatives and training programs to enhance students’ domain-specific knowledge and skills, thereby boosting their self-efficacy. Internet service providers should also prioritize platform usability and practicality. At the same time, they should strengthen guidance in areas such as interface design and security protection technologies. This would help students mitigate risks of information security and privacy threats without relying on overly complex operations.

College students’ response efficacy exhibits the most significant positive impact on their intention to protect information security and privacy. This may be attributed to the fact that when college students perceive that the information security protection behaviors are effective in safeguarding their personal information security, they will develop a positive attitude that such behaviors can bring practical security benefits, thereby strengthening their behavioral intentions towards information security protection. Conversely, if they perceive such protection behaviors as ineffective, they will form a negative attitude that these behaviors are meaningless, and their corresponding behavioral intentions will be suppressed (35, 61). This finding resonates with observations of global young adult populations, where the perceived effectiveness of protective actions consistently emerges as a key driver of information security behavior. This aligns with the core principles of PMT (33). In line with this finding, Mutchler et al. (62) have demonstrated that response awareness can enhance an individual’s intention to perform the secure behaviors by improving their self-efficacy. Lee et al. (63) have also found that the response efficacy of American college students regarding the use of virus protection software is an important influencing factor in developing virus protection intentions. In our survey, some respondents shared their views when answering items about obstacles to information protection. They answered, *“I installed antivirus software before but still encountered pop-up ads, so I thought it was ineffective and stopped using it afterward”* and *“The strong password I set could not keep hackers out; I figured it was useless to bother with, so I did not do it.”* These responses also show that when college students believe a certain information security behavior fails to reach their expected security goals, they will reject the security value of that behavior and give it up. They do this because they think their efforts will be pointless. Therefore, governments and universities should not only stress the importance of personal information security and privacy through public awareness campaigns but also highlight the key role of information security behavior in safeguarding such privacy. Simultaneously, efforts should be made to enhance students’ response efficacy and foster positive behavioral intentions to further encourage favorable actual behaviors. Additionally, network service providers should strengthen research on information security technologies to make network applications safer. In this way, they help build an optimal environment for college students’ information security and privacy.

The negative impact of college students’ response cost on information security privacy intention is significant. This indicates that college students’ response cost has negatively influenced their information security privacy intentions, which aligns with findings from related studies in the field of information security behavior (41). The result is further supported by research conducted by American scholars. From a cost–benefit perspective, Vishwanath et al. (64) studied how users protect their privacy on Facebook through a survey of American college students. They argue that users’ privacy protection behaviors in social networking services result from a trade-off between the accessibility of their accounts and the risk of personal information leakage. The study found that the cost of information disclosure and the benefit of information openness affect users’ privacy settings (64). The survey of Chinese college students also reveals that when implementing security measures, if such actions reduce user experience or impair service efficiency, students’ motivation to engage in secure behaviors may decrease. Specifically, on one hand, some online platforms restrict users’ access to certain or all features without getting prior authorization. Faced with such information collection and authorization requirements, some students may give up personal information security protection to avoid disruptions to their social activities. On the other hand, under heavy academic and social pressures, students not only need to spend time and energy learning various security measures but also deal with issues like software security scanning functions taking up device memory. Thus, when technical conditions are limited, many students choose to not implement safety measures to save resources or ensure their devices run smoothly. In our survey, some respondents pointed out obstacles to information protection, stating, *“Some genuine antivirus software requires payment, and security software may interfere with the regular operation of other software I use, causing significant inconvenience.”* and *“We frequently have to fill out personal information collection forms. It takes a lot of time to ask about the purpose of the information and whether we can skip filling it out every time.”* These responses reflect that the economic cost, convenience cost, time cost, and implicit energy cost associated with security protection measures will widen the gap between college students’ recognition of information security importance and their actual adoption of security behaviors. Consequently, enterprises should formulate simple, clear information security and privacy policies. They should maintain basic software functionality, minimize restrictive authorization demands on students, and give one-click safety tools or use cloud computing technologies to ease the pressure of data storage. These measures can effectively reduce the costs and burdens associated with students’ implementation of safety behaviors.

## 6 Conclusion

This study applies PMT to explore the factors influencing college students’ information security behavior and constructs a conceptual model for these behaviors. Empirical analysis shows that college students’ perceived threat, response efficacy, and self-efficacy all exert a positive influence on their personal information security behavior. In contrast, response cost exerts a negative influence on their protection intentions. Among these factors, response efficacy stands out as the most critical predictor.

This study holds substantial theoretical value. On the one hand, it takes PMT as the core framework and focuses on college students who are the core Internet user demographic. The study systematically integrates the threat appraisal and coping appraisal dimensions into the research on college students’ information security behavior. Through empirical analysis, it quantitatively clarifies the specific correlations between each dimension and information security behavior. It also identifies that response efficacy has the strongest positive effect. In doing so, the study provides empirical support for the application of PMT in segmented populations and emerging fields. On the other hand, combined with the context of rampant personal privacy breaches in the information economy era, the study adopts rigorous methods such as reliability tests, validity tests and regression analysis to validate the synergistic effect of the dual dimensions on information security behavior. This provides a referable variable framework and empirical basis for subsequent research, and promotes the deepening of the theoretical knowledge system in this field.

This study also yields substantial practical implications. The driving factors of college students’ information security behavior identified in this study provide actionable strategic insights for multiple subjects to implement information security governance. Among these factors, enhancing the effectiveness of information protection measures and reducing the cost of implementing such measures are particularly critical. Given that the study confirms response efficacy as a key driver of college students’ information security behavioral intentions, universities can design practical training programs based on the high-frequency information security scenarios in students’ daily lives. These programs will systematically teach students information security operation skills and theoretical knowledge. This will help students better perceive the effectiveness of protective measures and lay a solid foundation for developing positive information security behavior. Moreover, governments may encourage or require computer and software providers to develop user-friendly tools or informational prompts that remind college students to adopt protective behaviors. Enterprises should provide effective guarantees for students’ personal information security and privacy. They should prioritize such protection, foster a sound information security environment, and substantially reduce the temporal, technical, and economic costs students incur for protection. For their part, college students should learn more about information security skills and knowledge, develop daily protective habits, and raise their privacy awareness. When they find account abnormalities or information leakage, they should report the situation promptly through the university’s emergency hotline or the police service, turning information security protection into active behaviors in practice.

### 6.1 Limitation

This study used subjective self-reports to measure behavioral data, which limits data validity. Future studies could collaborate with university network centers or security software providers to obtain objective logs of college students’ information security behavior, cross-validate with self-reports, and boost measurement validity. They could also use controlled or scenario simulation experiments to directly observe students’ actual security behaviors. Combining subjective self-reports, objective logs, and experimental observations can more accurately verify the causal mechanisms of information security behavior via methodological complementation and corroboration. In addition, the sample of this study is limited to Chinese college students, and no cross-cultural comparative analysis is included, which means that the applicability of the research model in different cultural contexts has not been verified. To address this, future studies can expand into the field of cross-cultural comparison, covering samples of college students from different countries and cultural circles, and exploring the correlation mechanisms between variables in combination with cultural dimension theories. Furthermore, the research method is mainly quantitative analysis, with insufficient integration of qualitative research methods. This makes it difficult to deeply explain the underlying logic behind phenomena, thereby affecting the explanatory power and richness of the conclusions. In the future, a mixed research method combining quantitative surveys and qualitative interviews can be adopted, and in-depth interviews or case studies can be used to supplement the explanation of the internal mechanisms of variable relationships. What’s more, this study adopts a cross-sectional design, which limits the ability to infer causal relationships. Future studies using longitudinal or experimental designs will significantly enhance the validation of causal relationships.

## Data Availability

The datasets presented in this article are not readily available because the data used in this study is confidential and cannot be publicly shared. Requests to access the datasets can be directed to the corresponding author.

## References

[1] Cremer F Sheehan B Fortmann M Kia AN Mullins M Murphy F. Cyber risk and cybersecurity: a systematic review of data availability. Geneva Pap Risk Insur Issues Pract. (2022) 47:698–736. doi: 10.1057/s41288-022-00266-6, PMID: 35194352 PMC8853293

[2] Qin L Hui Y. Alienation and reconstruction of college students’ online civil literacy: based on a sampling survey of over 60,000 college students nationwide. J Northwest Normal Univ. (2025) 62:94–102. doi: 10.16783/j.cnki.nwnus.2025.02.010

[3] Department for Science, Innovation and Technology. Cyber security breaches survey (2023): Education institutions annex. GOV.UK. Available online at: https://www.gov.uk/government/statistics/cyber-security-breaches-survey-2023/cyber-security-breaches-survey-2023-education-institutions-annex (accessed on 30 August 2025).

[4] Ahmed M Kambam HR Liu Y Jaidka S Petrova K. Impact and significance of human factors in digital information security. Int J Inform Sci Technol. (2024) 7:1–17. doi: 10.57675/IMIST.PRSM/ijist-v7i2.213

[5] Chowdhury NH Adam MTP Teubner T. Time pressure in human cybersecurity behavior: theoretical framework and countermeasures. Computers Secur. (2020) 97:101963. doi: 10.1016/j.cose.2020.101963

[6] Zhang Z Zhu L. Intelligent detection and defense against adversarial content evasion: a multi-dimensional feature fusion approach for security compliance. Spectrum Res. (2024) 4:1–21. Available at: https://spectrumofresearch.com/index.php/sr/article/view/29

[7] Bekkers L van’t Hoff-De Goede S Misana-ter Huurne E van Houten Y Spithoven R Leukfeldt ER. Protecting your business against ransomware attacks? Explaining the motivations of entrepreneurs to take future protective measures against cybercrimes using an extended protection motivation theory model. Comput Secur. (2023) 127:103099. doi: 10.1016/j.cose.2023.103099

[8] Farshadkhah S Van Slyke C Fuller B. Onlooker effect and affective responses in information security violation mitigation. Comput Secur. (2021) 100:102082. doi: 10.1016/j.cose.2020.102082

[9] Bax S McGill T Hobbs V. Maladaptive behaviour in response to email phishing threats: the roles of rewards and response costs. Comput Secur. (2021) 106:102278. doi: 10.1016/j.cose.2021.102278

[10] Wang C Liu L Xu C Lv W. Predicting future driving risk of crash-involved drivers based on a systematic machine learning framework. Int J Environ Res Public Health. (2019) 16:334. doi: 10.3390/ijerph16030334, PMID: 30691063 PMC6388263

[11] Alrawhani EM Romli A Al-Sharafi MA. Evaluating the role of protection motivation theory in information security policy compliance: insights from the banking sector using PLS-SEM approach. J Open Innov: Technol Mark Complex. (2025) 11:100463. doi: 10.1016/j.joitmc.2024.100463

[12] Lahiri A Jha SS Chakraborty A Dobe M Dey A. Role of threat and coping appraisal in protection motivation for adoption of preventive behavior during COVID-19 pandemic. Front Public Health. (2021) 9:678566. doi: 10.3389/fpubh.2021.678566, PMID: 34291027 PMC8287502

[13] Aizawa K. Cognition and behavior. Synthese. (2017) 194:4269–88. doi: 10.1007/s11229-014-0645-5

[14] Brand BM. Bridging the intention-behavior-gap through digitalized information-two laboratory experiments in the textile industry. J Retail Consum Serv. (2025) 84:104179. doi: 10.1016/j.jretconser.2024.104179

[15] Guo Y Lu Z Kuang H Wang C. Information avoidance behavior on social network sites: information irrelevance, overload, and the moderating role of time pressure. Int J Inf Manag. (2020) 52:102067. doi: 10.1016/j.ijinfomgt.2020.102067

[16] Ajzen I. The theory of planned behavior: frequently asked questions. Hum Behav Emerg Technol. (2020) 2:314–24. doi: 10.1002/hbe2.195

[17] Martens M De Wolf R De Marez L. Investigating and comparing the predictors of the intention towards taking security measures against malware, scams and cybercrime in general. Comput Human Behav. (2019) 92:139–50. doi: 10.1016/j.chb.2018.11.002

[18] Mubarkoot M Altmann J Rasti-Barzoki M Egger B Lee H. Software compliance requirements, factors, and policies: a systematic literature review. Comput Secur. (2023) 124:102985. doi: 10.1016/j.cose.2022.102985

[19] Alam SS Ahsan N Kokash HA Alam S Ahmed S. A students’ behaviors in information security: extension of protection motivation theory (PMT). Inf Secur J Glob Perspect. (2025) 34:191–213. doi: 10.1080/19393555.2024.2408264

[20] Mousavi R Chen R Kim DJ Chen K. Effectiveness of privacy assurance mechanisms in users' privacy protection on social networking sites from the perspective of protection motivation theory. Decis Support Syst. (2020) 135:113323. doi: 10.1016/j.dss.2020.113323

[21] Mehraj H Jayadevappa D Haleem SLA Parveen R Madduri A Ayyagari MR. Protection motivation theory using multi-factor authentication for providing security over social networking sites. Pattern Recogn Lett. (2021) 152:218–24. doi: 10.1016/j.patrec.2021.10.002

[22] Zhang Z Zhang X. Why not use facial recognition payment? From the perspective of the extended protection motivation theory. J Retailing Consum Serv. (2024) 81:104016. doi: 10.1016/j.jretconser.2024.104016

[23] Tatiana B Kobicheva A Tokareva E Mokhorov D. The relationship between students’ psychological security level, academic engagement and performance variables in the digital educational environment. Educ Inf Technol. (2022) 27:9385–99. doi: 10.1007/s10639-022-11024-5, PMID: 35370438 PMC8964386

[24] Ng TK. New interpretation of extracurricular activities via social networking sites: a case study of artificial intelligence learning at a secondary school in Hong Kong. J Educ Train Stud. (2020) 9:49. doi: 10.11114/jets.v9i1.5105

[25] Ismail A Rose IR Foboy NA. Service quality as an antecedent in enhancing customers behavioural intentions: A case study of Malaysian army medical centers. Malaysian Journal of Society and Space. (2017) 12:179–90.

[26] Rogers RW. A protection motivation theory of fear appeals and attitude change1. J Psychol. (1975) 91:93–114. doi: 10.1080/00223980.1975.9915803, PMID: 28136248

[27] Mou J Cohen JF Bhattacherjee A Kim J. A test of protection motivation theory in the information security literature: a meta-analytic structural equation modeling approach. J Assoc Inf Syst. (2022) 23:196–236. doi: 10.17705/1jais.00723

[28] Gerdenitsch C Wurhofer D Tscheligi M. Working conditions and cybersecurity: time pressure, autonomy and threat appraisal shaping employees’ security behavior. Cyberpsychol J Psychosoc Res Cyberspace. (2023) 17. doi: 10.5817/CP2023-4-7

[29] Verkijika SF. Understanding smartphone security behaviors: an extension of the protection motivation theory with anticipated regret. Computers Sec. (2018) 77:860–70. doi: 10.1016/j.cose.2018.03.008

[30] Tsai HS Jiang M Alhabash S LaRose R Rifon NJ Cotton SR. Understanding online safety behaviors: a protection motivation theory perspective. Comput Secur. (2016) 59:138–50. doi: 10.1016/j.cose.2016.02.009

[31] Ng KC Zhang X Thong JYL Tam KY. Protecting against threats to information security: an attitudinal ambivalence perspective. J Manage Inf Syst. (2021) 38:732–64. doi: 10.1080/07421222.2021.1962601

[32] Wang H W Kuo S Y Chen L B. Exploring the relationship between internal information security, response cost, and security intention in container shipping. Appl Sci. (2021) 11:2609. doi: 10.3390/app11062609

[33] Liang H Xue Y. Understanding security behaviors in personal computer usage: a threat avoidance perspective. J Assoc Inf Syst. (2010) 11:394–413.

[34] Djatsa F. Threat perceptions, avoidance motivation and security behaviors correlations. J Inf Secur. (2019) 11:19.

[35] Klobas JE McGill T Wang X. How perceived security risk affects intention to use smart home devices: a reasoned action explanation. Comput Secur. (2019) 87:101571. doi: 10.1016/j.cose.2019.101571

[36] Anderson CL Agarwal R. Prating safe computing: a multimedia empirical examination of home computer user security behavioral intentions. MIS Q. (2010) 34:613–43. doi: 10.2307/25750694

[37] Hoegen PA De Bot CMA Echteld MA Vermeulen H. Measuring self-efficacy and outcome expectancy in evidence-based practice: a systematic review on psychometric properties. Int J Nursing Studies Adv. (2021) 3:100024. doi: 10.1016/j.ijnsa.2021.100024, PMID: 38746727 PMC11080366

[38] Li H Yoo S Kettinger WJ. The roles of IT strategies and security investments in reducing organizational security breaches. J Manage Inf Syst. (2021) 38:222–45. doi: 10.1080/07421222.2021.1870390

[39] Ifinedo P. Information systems security policy compliance: an empirical study of the effects of socialisation, influence, and cognition. Inf Manag. (2014) 51:69–79. doi: 10.1016/j.im.2013.10.001

[40] Yoon C Hwang JW Kim R. Exploring factors that influence students Behaviors in information security. J Inf Syst Educ. (2012) 23:407–15. Available at: https://aisel.aisnet.org/jise/vol23/iss4/7

[41] Iyer A. Understanding advantaged groups' opposition to diversity, equity, and inclusion (DEI) policies: the role of perceived threat. Soc Personal Psychol Compass. (2022) 16:e12666

[42] Chauhan V. Understanding users' protective behavior and its suppressor effect on the perceived risk in M-wallet/banking use: an Indian urban-rural comparison. Technol Forecast Soc Change. (2024) 201:123255. doi: 10.1016/j.techfore.2024.123255

[43] Glatz T Lippold M Chung G Jensen TM. A systematic review of parental self-efficacy among parents of school-age children and adolescents. Adolesc Res Rev. (2024) 9:75–91. doi: 10.1007/s40894-023-00216-w

[44] Klyver K Steffens P Nielsen SL. Crisis response efficacy: perceived ability to respond entrepreneurially to crises. J Bus Ventur Insights. (2023) 20:e00429. doi: 10.1016/j.jbvi.2023.e00429

[45] Thompson N McGill TJ Wang X. “Security begins at home”: determinants of home computer and mobile device security behavior. Comput Secur. (2017) 70:376–91. doi: 10.1016/j.cose.2017.07.003

[46] Ali RF Dominic PDD Ali SEA Rehman M Sohail A. Information security behavior and information security policy compliance: a systematic literature review for identifying the transformation process from noncompliance to compliance. Appl Sci. (2021) 11:3383. doi: 10.3390/app11083383

[47] DeVellis RF Thorpe CT. Scale development: Theory and applications. California: Sage publications (2021).

[48] Kirchherr J Charles K. Enhancing the sample diversity of snowball samples: recommendations from a research project on anti-dam movements in Southeast Asia. PLoS One. (2018) 13:e0201710. doi: 10.1371/journal.pone.0201710, PMID: 30133457 PMC6104950

[49] Zhan H Zheng C Zhang X Yang M Zhang L Jia X. Chinese college students' stress and anxiety levels under COVID-19. Front Psych. (2021) 12:615390. doi: 10.3389/fpsyt.2021.615390, PMID: 34177635 PMC8222572

[50] Wu D Yu L Yang T Cottrell R Peng S Guo W. The impacts of uncertainty stress on mental disorders of Chinese college students: evidence from a nationwide study. Front Psychol. (2020) 11:243. doi: 10.3389/fpsyg.2020.00243, PMID: 32210868 PMC7075936

[51] Baltar F Brunet I. Social research 2.0: virtual snowball sampling method using Facebook. Internet Res. (2012) 22:57–74. doi: 10.1108/10662241211199960

[52] Israel G D. Determining sample size. Florida: University of Florida. (1992).

[53] Hair JF Black WC Babin BJ Anderson RE. Tatham multivariate data analysis: Pearson education. New Jersey: Pretince Hall (2010).

[54] Taber KS. The use of Cronbach’s alpha when developing and reporting research instruments in science education. Res Sci Educ. (2018) 48:1273–96. doi: 10.1007/s11165-016-9602-2

[55] Al-Hashem N Saidi A. The psychological aspect of cybersecurity: understanding cyber threat perception and decision-making. Int J App Machine Learn Comput Intell. (2023) 13:11–22.

[56] Öğütçü G Testik ÖM Chouseinoglou O. Analysis of personal information security behavior and awareness. Comput Secur. (2016) 56:83–93. doi: 10.1016/j.cose.2015.10.002

[57] Chen Y Zahedi FM. Individuals’ internet security perceptions and behaviors. MIS Q. (2016) 40:205–22. Available at: https://www.jstor.org/stable/26628390

[58] Chang HH Wong KH Lee HC. Peer privacy protection motivation and action on social networking sites: privacy self-efficacy and information security as moderators. Electron Commerce Res Appl. (2022) 54:101176. doi: 10.1016/j.elerap.2022.101176

[59] Zhang M Nazir MS Farooqi R Ishfaq M. Moderating role of information asymmetry between cognitive biases and investment decisions: A mediating effect of risk perception. Front Psychol. (2022) 13:828956. doi: 10.3389/fpsyg.2022.828956, PMID: 35391971 PMC8982708

[60] Bottemanne H Morlaàs O Fossati P Schmidt L. Does the coronavirus epidemic take advantage of human optimism bias? Front Psychol. (2020) 11:2001. doi: 10.3389/fpsyg.2020.02001 32982839 PMC7479219

[61] Sommestad T Karlzén H Hallberg J. The theory of planned behavior and information security policy compliance. J Comput Inf Syst. (2019) 59:344–53. doi: 10.1080/08874417.2017.1368421

[62] Mutchler Leigh A. Response awareness and instructional self-efficacy: Influences on intent. Inform Computer Sec, (2019), 27: 489–507. doi: 10.1108/ICS-05-2018-0061

[63] Lee D Larose R Rifon N. Keeping our network safe: a model of online protection behaviour. Behav Inform Technol. (2008) 27:445–54. doi: 10.1080/01449290600879344

[64] Vishwanath A Xu W Ngoh Z. How people protect their privacy on Facebook: a cost-benefit view. J Assoc Inf Sci Technol. (2018) 69:700–9. doi: 10.1002/asi.23894

